# The granuloma in cryptococcal disease

**DOI:** 10.1371/journal.ppat.1009342

**Published:** 2021-03-18

**Authors:** Laura C. Ristow, J. Muse Davis

**Affiliations:** Stead Family Department of Pediatrics, University of Iowa Carver College of Medicine, Iowa City, Iowa, United States of America; Vallabhbhai Patel Chest Institute, INDIA

## Abstract

Although we have recognized cryptococcosis as a disease entity for well over 100 years, there are many details about its pathogenesis which remain unknown. A major barrier to better understanding is the very broad range of clinical and pathological forms cryptococcal infections can take. One such form has been historically called the cryptococcal granuloma, or the cryptococcoma. These words have been used to describe essentially any mass lesion associated with infection, due to their presumed similarity to the quintessential granuloma, the tubercle in tuberculosis. Although clear distinctions between tuberculosis and cryptococcal disease have been discovered, cellular and molecular studies still confirm some important parallels between these 2 diseases and what we now call granulomatous inflammation. In this review, we shall sketch out some of the history behind the term “granuloma” as it pertains to cryptococcal disease, explore our current understanding of the biology of granuloma formation, and try to place that understanding in the context of the myriad pathological presentations of this infection. Finally, we shall summarize the role of the granuloma in cryptococcal latency and present opportunities for future investigations.

## Historical perspectives: The granuloma as mass

The earliest descriptions and summaries of cryptococcal infection (then called torulosis) posed the disease as a less common and slower moving relative of tuberculosis [1]. A perceived propensity for forming tumor-like masses in the lungs and brain gave rise to the name “neoformans,” which has haunted the evolving nomenclature ever since. As our understanding of this infection improved, it became clear that such masses were not exceptionally common overall. Still, such lesions, often called granulomas or cryptococcomas, became closely associated with the pathology of the disease.

Descriptions of these lesions are varied—in terms of size, location, number, and amount of inflammation present. Multiple authors, beginning with Cox and Tolhurst in 1946 [2], emphasized a dichotomy between “gelatinous” versus “fibrotic or granulomatous” masses. The former could be found in both lung and brain, among other tissues, and consisted mostly of large numbers of replicating yeast. Although these were usually demarcated and distinct from the surrounding tissue, inflammatory cells were scarce.

We cannot know to what extent the subsequently distinguished species, *Cryptococcus gattii*, represented the infectious agent in these early Australian studies. However, it is worth considering that *C*. *gattii* is much more prone to generating the gelatinous form of lesions [3]. “Fibrotic or granulomatous” masses, on the other hand, were primarily found in the lung and were frequently compared to tubercles, the characteristic gross pathologic feature of tuberculosis.

In 1955, Baker and Haugen [4] made an interesting observation about duration of infection and pathology. Cases less than 8 weeks in duration (most likely rapidly evolving) often featured gelatinous masses with minimal inflammation. On the other hand, cases developing over 8 weeks to 2 years developed masses with fewer organisms and more often featured granulomatous lesions. Frequently, clinical case reports referred to both of these lesion types as “cryptococcal granulomata.” Baker and colleagues later cemented the case for a “true” cryptococcal granuloma by correlating subpleural nodules, sometimes in association with an infected lymph node, with the earliest stages of cryptococcal disease [5]. They proposed that the subpleural nodule was the primary site of infection; in most people, it gradually resolves or contains infection indefinitely—not at all unlike tuberculosis.

## What is a granuloma?

This word has a long and poorly understood history. The gross pathological structure was first described in 1679, referring to the nodular “tubercles” of tuberculosis [6]. The eminent Rudolf Virchow is credited with coining the term “granuloma” in 1863 for what he considered to be tumors of granulation tissue similar in appearance to bone marrow [7]. Granulation tissue at that point was a term with only gross pathological meaning. The lesions of tuberculosis were still the quintessential example of granulomas, although for Virchow tuberculosis included among its forms several disease states now known to be distinct, ranging from rheumatological to neoplastic. Still, the origins of the term have led to its use for many kinds of masses regardless of their origin.

**Fig 1:**
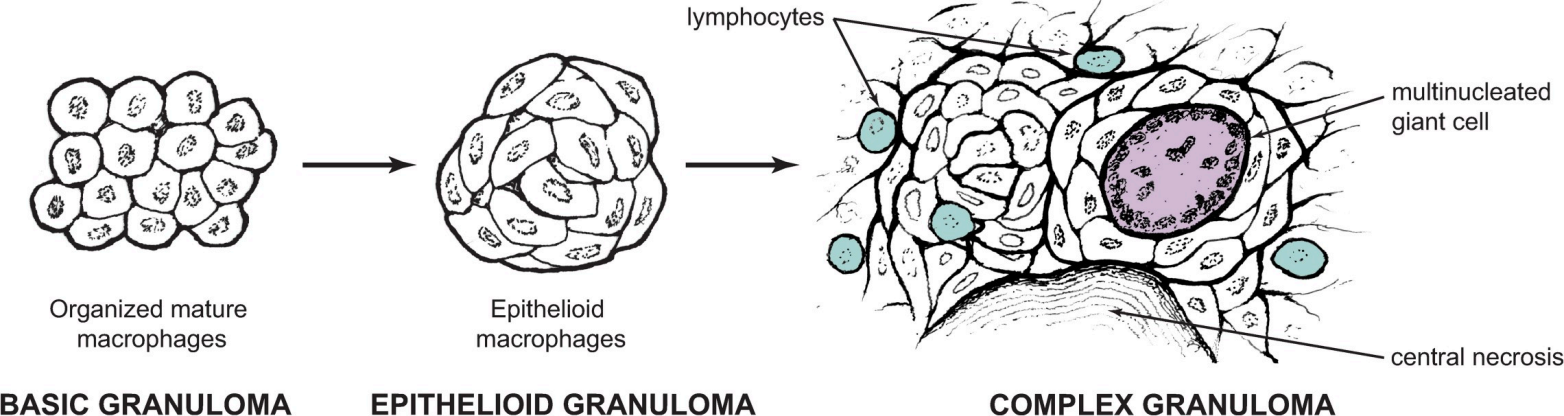
Fundamental granuloma types. According to Adams [8], a basic granuloma need only contain organized mature macrophages in the context of chronic inflammation (left). Depending on the inciting stimulus, this arrangement may progress to include epithelioid transformation of these macrophages (middle), with formation of tightly joined interdigitated cell membranes. Several other features may subsequently appear in a complex granuloma (right), including central necrosis, multinucleated giant cells, and lymphocytes. Other “accessory” features not pictured include other leukocytes such as neutrophils or eosinophils, B and T cells, and fibrosis [10]. Image partially redrawn from [8].

For a reckoning of the essential properties of granulomatous inflammation in a cellular and molecular sense, we must look far forward in time to the 1960s and 1970s, when the question of the overarching biology of granulomatous inflammation was separately summed up by 2 researchers, Dolph O. Adams [8] and Dov L. Boros [9]. For Adams, the essential feature was an organized collection of mature macrophages, possibly accompanied by epithelioid transformation, central necrosis, and other cell types appearing as optional, secondary features (Fig 1). This definition notably considers adaptive immunity to be involved but not required. Boros supported the idea of a granuloma as a “focal, chronic inflammatory response to tissue injury evoked by a poorly soluble substance,” leaving room to consider the adaptive immune system a prerequisite. Key to both of these is the concept of a conserved multicellular program which appears in many different contexts but has its own developmental pattern. Indeed, examination of organisms as disparate as humans and flies suggests an evolutionarily ancient system of phagocyte cooperation in the face of a chronic stimulus [10].

More recent research has given new life to Adams’ proposed “phagocytes-first” definition. A molecular program for the early stages is beginning to take shape, starting with the process of epithelioid transformation of macrophages. The up-regulation of E-cadherin by macrophages is a prominent molecular milestone, induced within days of infection in a zebrafish model of tuberculosis [11]. Upstream of this process appears to be STAT6 signaling triggered by Th2 cytokines, observed in schistosomiasis [12]. One molecular trigger for this process prior to the involvement of adaptive immunity (as in Adams’ organized collection of mature macrophages) is the kinase mammalian target of rapamycin complex (mTORC), the chronic activation of which induces epithelioid transformation in murine macrophages [10]. Of course, according to Adams, epithelioid transformation is not the initial step in granuloma formation, so many more early details remain to be worked out.

Another key question of granuloma biology is how the ultimate fate of the lesion is determined. In broad strokes, granulomas can (a) shrink and recede, (b) grow and become a persistent nodular lesion, often with fibrosis or calcification, or (c) fail to sustain their structure and give way to the growth of a pathogen or tumor [10]. These later “developmental” steps are very much under the influence of adaptive immunity and are better understood. In general, the cytokine milieu, as a product of the adaptive immune response (for example, Th1 versus Th2), is the key driver, although our understanding of how and when these responses mature is still under active study.

### Pathology of the cryptococcal granuloma and granulomatous inflammation

**Fig 2:**
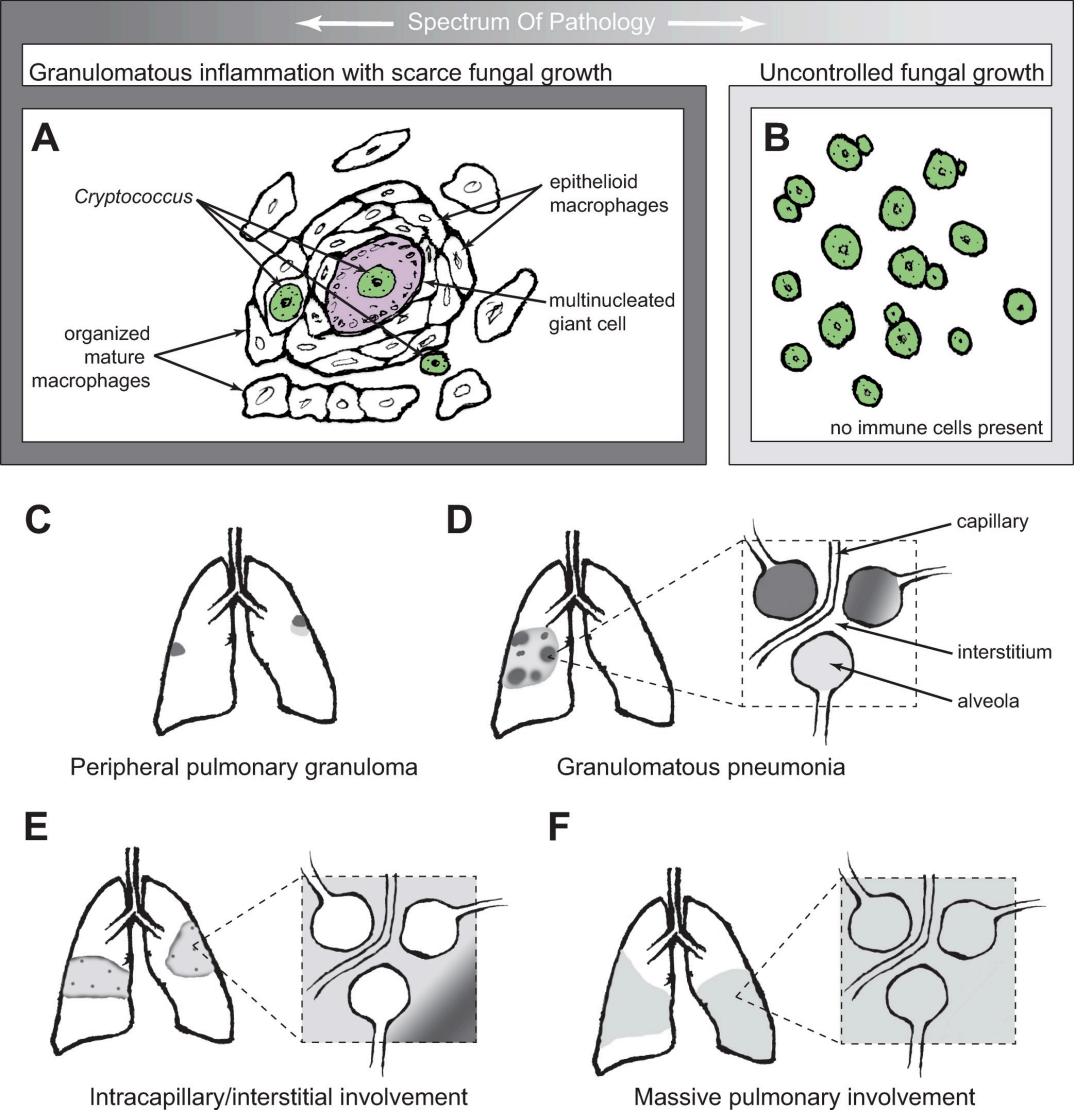
Pathological types of pulmonary cryptococcosis. Panels A and B represent the extreme ends of a spectrum of pathological findings. This spectrum is represented differently in the 4 pathological types (C–F). (A) At one end of the spectrum, granulomatous inflammation featuring the pictured elements is capable of limiting cryptococcal growth. Dark gray frame color indicates location of these findings in subsequent panels. (B) At the other end of the spectrum is uncontrolled fungal growth with minimal or no inflammatory response. Light gray frame color indicates location of these findings in subsequent panels. (C) Peripheral pulmonary granuloma may consist entirely of granulomatous inflammation (dark gray only, left), or may have areas with emerging fungal growth (lighter gray adjacent to dark gray, right). (D) Granulomatous pneumonia, with patchy granulomatous response amid fungal growth entirely within alveoli. The patchiness of the overall picture (left) represents intra-alveolar pathology representing different parts of the spectrum. (E) Intracapillary/interstitial involvement, in which the alveoli are not involved. Patches of granulomatous inflammation are smaller and more dispersed, and pathology at varying points on the spectrum is seen only in the interstitial space. (F) Massive pulmonary involvement, with uncontrolled fungal growth in all areas.

As noted above, at the gross pathology level, a dichotomy has been proposed between “gelatinous” and “fibrotic or granulomatous” cryptococcal masses in the lung [4]. On histological examination, in the latter, there were usually far more inflammatory cells than yeast, and indeed, macrophages, epithelioid cells, and giant cells are described repeatedly in these cases [4] (Fig 2A). While some gelatinous lesions feature almost no inflammation whatsoever (Fig 2B), closer review suggests that there is a spectrum of inflammatory involvement in such lesions. In 1985, McDonnell and Hutchins [13] detailed a series of 36 autopsy studies in which cryptococcosis was found. They describe 4 categories of pathological findings:

#### Peripheral pulmonary granuloma (Fig 2C)

In 3 of the 36 cases, intact, “quiescent” lesions with scant fungi and prominent granuloma structure were found. Only one of these 3 had clinical findings of cryptococcal disease. Four cases showed similar lesions which appeared to be disrupted, with granulomatous inflammation in areas giving way to unchecked fungal growth. All of these patients had symptomatic disease. This category aligns well with Baker’s subpleural nodules [4].

#### Granulomatous pneumonia (Fig 2D)

This was the most common form found, with 19 cases. In these, diffuse fungal replication was seen in the alveoli, and the inflammatory reaction varied from none, with many yeast cells present, to the appearance of epithelioid macrophages and multinucleated giant cells, correlating with scarce fungal cells.

#### Intracapillary/interstitial involvement (Fig 2E)

Seven cases featured this form of pathology, in which the infection and response are in the interstitial spaces and not the alveoli. They report limited evidence of inflammation, except for occasional patches of “interstitial granulomas.” In one of these cases, the cryptococci were seen only in interstitial capillaries.

#### Massive pulmonary involvement (Fig 2F)

Three cases featured large amounts of intra-alveolar and intracapillary fungal growth, with essentially no evidence of an inflammatory response.

These categories describe a rough spectrum from quiescent granulomatous lesions, to lesions featuring both granulomatous inflammation and heavy fungal replication, finally to massive cryptococcal replication with virtually no inflammatory response. Thus, cryptococcal infection does incite the evolutionarily ancient process of granulomatous inflammation, and the less active it is, the more rapidly the fungus can replicate.

In the brain, diffuse meningoencephalitis is by far the most common pathology. When mass lesions do appear, they have historically been difficult to distinguish from neoplasms [14]. Pathologically they have mostly been described as featuring a prominent granulomatous response, but “cystic” lesions with cryptococcal growth surrounded by a fibrous capsule have also been reported [15]. Interpretation of historical accounts is complicated by the fact that *Cryptococcus neoformans* and *C*. *gattii* were not distinguishable until much more recently. Although both species can cause focal intracranial lesions, *gattii* appears to be much more predisposed to this [16]. It has been suggested that overall *gattii* pathology is more likely to involve a granulomatous response [17], but due to the wide variations in pathology of cryptococcosis in general, this is difficult to confirm. A more firm observation is that *C*. *gattii* granulomas contain more T cells and can even play host to follicular structures of B and T cells [17]. This is in line with the tendency of *C*. *gattii* to affect immunocompetent hosts.

The subpleural nodule described by Baker and referred to by McDonnell and Hutchins as a peripheral pulmonary granuloma most closely matches the classic description of tuberculosis [4,13]. It has long been held that the TB granuloma is a static entity, resolutely “walling off” an infection that cannot be eradicated, until some unknown event triggers its reemergence [18]. More recent work has demonstrated that these isolated lesions are in fact quite dynamic, allowing the influx and efflux of both host and pathogen cells, even providing a “safe haven” for mycobacteria [10]. Indeed, for tuberculosis, the earliest stages of granuloma formation are subverted to enhance mycobacterial replication. In contrast, it seems that cryptococcal replication is only hampered by the granuloma response. The varying pathological findings in cryptococcal infection, with fungal growth seen in inverse proportion to granulomatous inflammation, is highly suggestive of a situation in which the granulomatous response must either fail at the start or erode over time in order for clinical disease to occur.

A particularly fertile avenue for studying this question in mice has been established using a cryptococcal strain lacking functional glucosylceramide synthase 1 (GCS1) [19]. This strain grows poorly in the extracellular environment, presumably due to poor tolerance of neutral to alkaline pH and elevated CO_**2**_. Mice infected with this strain via the airway do not develop disseminated infection, but rather produce well-defined granulomas in the lungs, which contain small numbers of yeast cells [19]. Further research has shown that host-derived sphingosine kinase 1 (SK1) is required for this protective response to infection with *Δgcs1* yeast [20]. Sphingosine 1-phosphate (S1P), a product of SK1, has multiple roles in innate immune cell signaling, and its interaction with S1P receptor 2 appears to be central to the granulomatous response induced by *Δgcs1* [21,22]. Changes in cytokine expression, including monocyte chemoattractant protein 1 (MCP1) and tumor necrosis factor alpha (TNF-α), are part of the effect. Exactly where this particular mechanism of granuloma induction fits within the pathogenesis of human cryptococcosis, and into the evolutionarily ancient granuloma developmental process, remains to be seen.

## The granuloma in cryptococcal latency

Latent infection is an ill-defined phenomenon for *Cryptococci*. Here we will consider it to mean the host–pathogen relationship that exists between initial infection and clinical disease. Because a primary cryptococcal infection can so often be subclinical, the true role of the latent period in overall disease burden is hard to characterize. Regardless, there is evidence for reactivated latent infections due to *C*. *neoformans*. One of the first reports to describe a link between primary and reactivated infection biochemically demonstrated high serum reactivity to cryptococcal proteins prior to solid organ transplantation in patients who ultimately were diagnosed with a cryptococcal infection following transplantation [23]. In several studies, researchers characterized genomic relatedness of patient and environmental isolates, concluding that there were many examples of acute infections caused by strains that were typical of the patient’s country of origin rather than their place of residence [24,25]. In some reports, the patients hadn’t been to their country of origin for years [24,25]. Although case studies are numerous for suspected reactivation of infection with *C*. *neoformans*, there are only 2 published reports of reactivated latent infection attributed to *C*. *gattii* [26,27], highlighting another difference in the pathophysiology of these distinct species.

Latent or persistent infection by *C*. *neoformans* is experimentally supported in animal models of cryptococcal infection, as low-level cryptococcosis can persist in the rat or mouse lung following primary infection. The canonical cryptococcal latency investigation utilized the biologically relevant inhalation model and examined cortisone treatment effects on pulmonary cryptococcosis [28]. In the absence of immune suppression via cortisone treatment, rats and guinea pigs survived infection with a sublethal dose of *C*. *neoformans*. Strikingly, if cortisone was administered less than 4 weeks following the experimental inoculation of *C*. *neoformans*, 55%–90% of rats were overcome by fungal disease [28]. In the rat model, cryptococcal infection mimics the hypothesized course of latent infection in humans with persistence of yeast within macrophages and epithelial cells, as well as granuloma formation within the rat lung [29]. Revisiting the canonical study at a microscopic level, corticosteroid administration can reactivate infection in a latently infected rat from the intracellular residence within macrophages, or the granuloma to extracellularly detectable fungi [29]. In the mouse model, following a primary infection, low levels of *C*. *neoformans* are detectable before and during a second infection induced with a new bolus of *C*. *neoformans* [30]. The authors hypothesize that the low levels of yeast present throughout the experimental protocol in the lungs represent the source of reactivated infection upon immune suppression in humans [30]. A subpopulation of yeast cells is present within the mouse lung with a phenotype consistent with dormant or latent yeast, including low metabolic activity and a delay in, or inability to reenter exponential growth once recovered from mice or in vitro macrophages [31]. Both rodent models provide evidence for latent infection and granuloma formation as a part of the natural course of a *C*. *neoformans* infection [5,29,32].

The granuloma is a very plausible niche for persistent cryptococci to reside in, especially since these lesions can be found incidentally in humans, in the absence of disease [4]. In patients with active disease, subpleural nodules are present upon X-ray in the lungs, reminiscent of tuberculosis infection or lung cancer [33–35]. Exposure to cryptococci can occur early in life, as studies have reported as high as 63% of children from the Bronx over the age of 2 demonstrated reactivity to cryptococcal proteins, although the risk of disease resulting from primary exposure to cryptococci seems fairly low in immunocompetent individuals [36,37]. Depending on the incidence of primary cryptococcal infection resulting in latency, the threat of reactivation could affect significantly more individuals as survival with immunosuppressive infections or diseases and immunosuppressive therapies become more routine. Recent reports have associated the multiple sclerosis treatment, Fingolimod (FTY720), with a higher risk of cryptococcal infection, potentially due to reactivated latent infection [38,39]. The connection makes biological sense as FTY720 is converted into an analog of SP1 (see previous section) [40]. Given the importance of granulomatous inflammation in a protective host response, the better we can understand the cryptococcal granuloma, the better we may be able to anticipate and avoid reactivating disease.

## Key points and future directions

In this review, we have sought to condense a large number of observations into a unifying view of the role of granulomatous inflammation in cryptococcal disease. Main points to reinforce include:

1. The term “cryptococcal granuloma” has been historically overused to describe any mass lesion associated with the fungus. In truth, these mass lesions are heterogeneous and can contain plentiful granulomatous inflammation, or essentially none.
2. The current view of granuloma biology describes an evolutionarily ancient intercellular reaction which originates with phagocytes and can develop into more complex structures over time. Other cell types, including adaptive immune cells, can later become involved in and even required for granuloma integrity. This requirement explains the association between adaptive immune compromise and *C*. *neoformans* disease.
3. Granulomatous inflammation is characteristic and essentially required for control (and eradication?) of cryptococcal infection. The range of pathological findings shows that granuloma formation and cryptococcal replication appear in inverse proportions.
4. Subpleural granulomas, with or without their associated lymph nodes, are the likely site of quiescent survival by *C*. *neoformans*. In the immunocompetent host, these appear to form in the context of asymptomatic infection. In most cases, these likely eradicate the infection and resolve.
5. *C*. *gattii* differs from *C*. *neoformans* in being a more potent driver of granulomatous inflammation. This results in a predilection for clinical disease in immune competent hosts, who generally eradicate the infection over time.

A better understanding of the cryptococcal granuloma is essential for our efforts toward understanding initial infection, modulating the immune response toward low-morbidity outcomes, and predicting or even preventing reactivation. The following are some of the key questions for future research:

1. What determines the fate of a granuloma in initial cryptococcal infection? It appears that many immunocompetent hosts eradicate infection outright, while a smaller group may maintain a quiescent infection indefinitely. Based on epidemiology, the adaptive immune system (T cells in particular) clearly plays a major role. Still, growing evidence suggests that the granuloma at its earliest stages is a product of macrophage function alone [10]. The transition period from a purely innate stage to one that requires T cells is a major blind spot in our understanding. We need to adapt our models to explore better the complex and continuous interactions between adaptive and innate immunity. New three-dimensional in vitro approaches involving multiple cell types could be very useful for this purpose [41]. Also, the larval zebrafish model, a very useful tool for studying innate immunity, can be taken out to later stages in which adaptive immunity is present [11].
2. How does granulomatous inflammation maintain quiescence? After the granuloma is established, the next puzzle is how a stalemate between host and pathogen can be maintained for so long. Learning the specific pathways involved could lead to new, host-centered approaches for bypassing this requirement temporarily to maintain quiescence during periods of immunocompromise. Using the Δ*gcs1* strain in the context of mutant mouse models lacking components of the innate and adaptive immune response could help to identify critical components that maintain the cryptococcal organisms in check.
3. Where do the findings with the Δ*gcs1* strain fit in to the spectrum of human disease? It has been suggested that some cryptococcal strains are more prone to granuloma formation than others [42]. Investigation of such clinical strains, along with mutants such as Δ*gcs1*, using whole genome sequencing and newer gene expression assays directed at both host and pathogen, could offer a trove of new information about granulomatous inflammation in general and the cryptococcal granuloma in particular.
4. Why is *C*. *gattii* so much more effective at inducing granulomatous inflammation? Is this why *C*. *gattii* almost never produces quiescent infection? Is this relevant to the range of presentations and pathology we see in *C*. *neoformans*? In one relatively recent study, macrophage phagocytosis rates and gene expression patterns were shown to differ significantly between these 2 infections [43]. Further comparative studies of T cell response, dissemination, and, of course, granuloma formation, should provide many insights into the pathogenesis of both organisms.
